# A Tape-Wrapping Strategy towards Electrochemical Fabrication of Water-Dispersible Graphene

**DOI:** 10.3390/nano14090805

**Published:** 2024-05-06

**Authors:** Deyue Xiao, Peng He, Haolong Zheng, Shujing Yang, Siwei Yang, Guqiao Ding

**Affiliations:** 1 National Key Laboratory of Materials for Integrated Circuits, Shanghai Institute of Microsystem and Information Technology, Chinese Academy of Sciences, 865 Changning Road, Shanghai 200050, China; xiaodeyue21@mails.ucas.ac.cn (D.X.); zhenghaolong20@mails.ucas.ac.cn (H.Z.); yangsj@mail.sim.ac.cn (S.Y.); yangsiwei@mail.sim.ac.cn (S.Y.); 2 College of Materials Science and Opto-Electronic Technology, University of Chinese Academy of Sciences, Beijing 100049, China

**Keywords:** tape-trapping strategy, water dispersibility, electrochemical oxidation, exfoliation mechanism

## Abstract

Graphene has achieved mass production via various preparative routes and demonstrated its uniqueness in many application fields for its intrinsically high electron mobility and thermal conductivity. However, graphene faces limitations in assembling macroscopic structures because of its hydrophobic property. Therefore, balancing high crystal quality and good aqueous dispersibility is of great importance in practical applications. Herein, we propose a tape-wrapping strategy to electrochemically fabricate water-dispersible graphene (w-Gr) with both excellent dispersibility (~4.5 mg/mL, stable over 2 months), and well-preserved crystalline structure. A large production rate (4.5 mg/min, six times faster than previous electrochemical methods), high yield (65.4% ≤5 atomic layers) and good processability are demonstrated. A mechanism investigation indicates that the rational design of anode configuration to ensure proper oxidation, deep exfoliation and unobstructed mass transfer is responsible for the high efficiency of this strategy. This simple yet efficient electrochemical method is expected to promote the scalable preparation and applications of graphene.

## 1. Introduction

Graphene, as one of the most promising two-dimensional nanomaterials, has been developed in wide application fields such as energy generation and storage, biomedicine and electronic information [1,2,3,4,5,6]. This rapid development depends largely on various viable strategies demonstrated not only to prepare graphene materials, but also to regulate their chemical structure, dimensions and properties [7]. The main exfoliation methods of graphene include mechanical exfoliation, liquid phase exfoliation, oxidation–exfoliation–reduction and electrochemical exfoliation [8]. The discovery of graphene originated from micromechanical exfoliation [9].Furthermore, graphite sheets can be prepared with the help of mechanical equipment such as ball mills, but the obtained products are easy to aggregate and possess non-uniformity, which makes it difficult to meet the scale production needs of high-quality few-layer graphene. Mechanical effects also play a major role in the liquid phase exfoliation, and one of the most direct and effective way to reduce the strength of van der Waals attractions is liquid immersion [10]. However, most liquid phase exfoliation methods rely on “good” solvents such as N, N-Dimethylformamide (DMF) and N-Methyl pyrrolidone (NMP) to isolate graphene sheets with high crystal quality and obtain processability [11]. These high-boiling-point organic solvents were selected, rather than green, cost-effective and easy-to-remove water, because their surface energy matches well with the hydrophobic graphene [12].

Among various preparative routes, electrochemical methods stand out because of their evidenced high efficiency, greenness and potential scalability [13,14,15]. Chunlei Wang et al. developed a modified bipolar electrochemistry (BPE) approach to exfoliate graphite and prepare few-layer graphene [16], which further improved the electrochemical performance of graphene-based materials set in supercapacitor applications. Lots of efforts focusing on the design of the electrolyte systems, anode configurations and the pre-treatment of anode have greatly increased the controllability of crystal quality, doping and yield [17,18]. However, few studies have induced the dispersion of graphene in water despite its well-known advantages in practical applications. In the previous reports, graphene oxide (GO) has been successfully prepared with high yield (~90%) through different electrochemical strategies of oxidation enhancement [19,20,21]. As with conventional GO fabricated by the deep oxidation of graphite, this electrochemically derived GO possesses excellent dispersibility in water, but also is destructed seriously in its crystal structure, and requires special reduction processes for structure restoration. It is of great importance to give graphene good water dispersibility on the basis of no excessive damage to the *sp*^2^ domain of graphene. Recently, the electrochemical route was demonstrated to be greener and more viable to balance the water dispersibility and crystal structure compared with the conventional chemical oxidation routes. Our previous work [22,23] reported the molecule-based anode coverage strategy for electrochemical preparation of water-dispersible graphene (w-Gr) that exhibited processability comparable to GO. More importantly, the *sp*^2^ structure of w-Gr can be restored under mild thermal treatment, instead of the tedious and sometimes toxic chemical processes or high-temperature annealing that is necessary for GO reduction. However, all these electrochemical methods for preparing water-dispersible and easy-to-reduce graphene sheets suffer from very low production rates (a maximum of about 0.7 mg/min), which greatly limits their scalability.

Herein, we develop a tape-wrapping strategy to ensure proper oxidation of a graphite anode and electrochemically prepare w-Gr. The optimized anode configuration gives a high yield (65.44% for 1–5 atomic layer graphene) for w-Gr with excellent dispersibility (up to 4.5 mg/mL), good processability and reasonably high conductivity (11793 S/m after 200 °C thermal reduction). Mechanism analysis highlights that the rational tape-wrapping configuration of the anode is crucial to ensuring a continuous electrochemical process of graphite in a confined space and obtaining a high production rate and yield.

## 2. Materials and Methods

### 2.1. Chemicals

Graphite foil (0.55 mm, 99.8%) was purchased from Thermo Fisher Scientific (Waltham, MA, USA). Ammonium sulfate ((NH_4_)_2_SO_4_, AR) was purchased from Aladdin (Shanghai, China). Tape was purchased from Shanghai Titan Technology Corporation (Shanghai, China). The deionized water (DI water) used throughout the experiments was purified by a Millipore purification system (LD, Mighty-10). All materials and chemical reagents were used without further purification.

### 2.2. Preparation of w-Gr

The electrochemical DC power (KEYSIGHT N5765A) and reaction equipment are displayed in Figure S1a. The cooling water circulator (JULABO, F25) was used to control the system temperature. Graphite plate (1.5 cm × 10 cm) was used as a cathode and the graphite foil (1.5 cm × 10 cm) was configurated as an anode, respectively. The two electrodes were vertically inserted into the electrolyte ((NH_4_)_2_SO_4_, 1 M) with a distance of about 3 cm and applied with a constant voltage of 10 V at 25 °C for 2 h. The black product was washed 3 times by vacuum filtration in order to remove the residual salt solution. Ultrasonication in deionized water for 30 min by a bath sonication machine (JP-040ST, 500 W, 40 KHz) was conducted to further exfoliate the nanosheets. The sediment was removed after centrifugation (Thermo Fisher Scientific, Waltham, MA, USA, MUTIFUGE X1) at the rate of 15,000 rpm for 20 min, and a uniform dispersion was obtained. The stable dispersion was used for subsequent characterization and assembly into aerogels and films.

Anodes were designed through rational tape-wrapping configurations. The length and the width of the anodes involved in electrochemical reactions are 6 cm and 1.5 cm, respectively. The depth immersed in the electrolyte was 7.5 cm. 1# anode was wrapped by tape and folded with the open end upwards, and the section close to the open end (~4.4 cm) was equally divided into four segments (1.1 cm in length for each segment). 2# anode was directly exposed to electrolyte, and the tape was used only to ensure the same effective area as the other anodes. 3# anode was entirely wrapped by tape except the bottom side in contact with the electrolyte. 4# anode was similar to 3# anode, but it was folded to make the open bottom side upward. The photographs and three-dimensional model (Figures S1b,c and S2) show the configurations of these four anodes. 

### 2.3. Assembly of w-Gr

**Figure 1:**
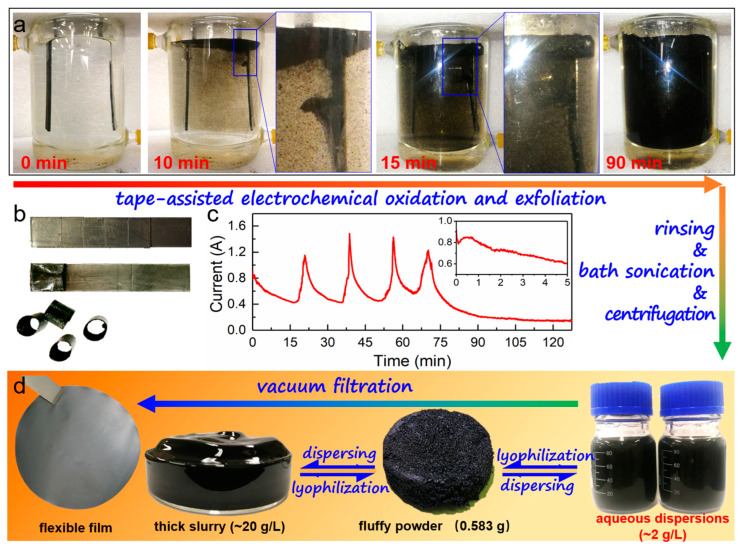
Preparation of water-dispersible graphene. (**a**) Process of electrochemical treatment for tape-wrapping strategy. (**b**) Change in 1# anode after electrochemical treatment. (**c**) Current–time curve of the electrochemical process; inset shows the details of the initial 5 min. (**d**) Preparative route of w-Gr for dispersions, powders, slurry and flexible film.

The fluffy porous aerogel (0.583 g) was prepared by 2 g/L w-Gr dispersion through lyophilization for 24 h using a freeze-drying machine (SCIENTZ-18N). The w-Gr powder can be redispersed in water to produce 20 g/L concentrated slurry. Graphene films can be prepared through vacuum filtration technology and the continuous centrifugation coating (CCC) method [24]. Typically, the w-Gr dispersion (2 mg/mL) was filtrated under vacuum through a 0.22 μm Millipore filter (Figure S3). Under vacuum-induced pressure, the w-Gr nanosheets in dispersion were uniformly deposited on the filter membrane. The filter membrane was made by CA-CN (cellulose acetate and cellulose nitrate) with a pore size of 0.22 μm and a filtering diameter of 6 cm in this system. Drying at 50 °C for 10 h and separation from the filter membrane yielded a free-standing and flexible w-Gr film (Figure 1d). The conductive film was synthesized using the CCC system (C^3^-100). The w-Gr dispersion (2 mg/mL) was continuously cast on the PET substrate to form a new film layer. The heating power was 300 W and the rotating rate was 2000 r/min. A free-standing w-Gr film (50 × 300 mm^2^) can be easily peeled off from the PET substrate after being taken out of the coating system. The obtained film was used for structure characterization and was thermally annealed at a low temperature (200 °C) for 1 h before the conductivity test. 

### 2.4. Characterization Methods

Scanning electron microscopy (SEM) images and energy-dispersive X-ray (EDX) spectroscopy were obtained by ZEISS Sigma 300. Atomic force microscopy (AFM) data was measured by a Bruker Multimode 8 in tapping mode. For SEM and AFM measurements, the 2 mg/mL aqueous dispersion was diluted and dropped onto the clean silicon oxide wafer and dried at 30 °C for 6 h for testing. For SEM-EDX analysis, the dried powder was placed and compacted on the electro-conductive adhesive tape. The microstructure and layer numbers of w-Gr were analyzed by Hitachi H-8100 high-resolution transmission electron microscopy (HR-TEM) at an accelerated voltage of 80 kV. The proper concentration of w-Gr dispersion was dropped onto the ultrathin carbon support film (Beijing Zhongjingkeyi Technology Co., Ltd., Beijing, China, BZ1103XX). After sample preparation, an infrared baking lamp (LP23030-B) was used for pre-drying treatment. Raman spectroscopy was carried out using a Renishaw inVia Reflex with a laser wavelength of 532 nm. X-ray diffraction (XRD) was carried out by the use of a D8 ADVANCE with Cu Kα1 radiation (λ = 0.1541 nm, Bruker, Germany) at 40 kV and 40 mA. Thermogravimetric analysis (TGA) curves were measured by a thermal analyzer (NETZSCH-Gerätebau GmbH, Selb, Germany, STA449F3, NETZSCH5) under a flow of argon kept at 20 mL/min. X-ray photoelectron spectroscopy (XPS) was carried out by using a Thermo ESCALAB 250XI. For Raman, XRD, TGA and XPS measurements, the samples to be tested were obtained by 2 mg/mL w-Gr dispersions through lyophilization for 24 h. Before Raman and XRD analysis, it was necessary to apply slight pressure to the powder sample for better imaging. A Fourier transform infrared spectrometry (FT-IR) result was obtained using a Bruker INVENI0-R, and the samples were prepared by mixing the w-Gr powder with KBr and pressing into a transparent pellet. The UV-vis spectrum was measured by an Agilent Technologies Cary 100. The zeta potential (ζ) was employed to quantitatively analyze the dispersity of w-Gr with a Zetasizer Nano-ZS90 (Malvern, UK). The 45 mg w-Gr powders obtained through lyophilization were dispersed in 10 mL deionized water to prepare a uniform dispersion with a concentration of 4.5 mg/mL, which was used for the above zeta potential testing. The conductivity of the graphene film was measured by the four-point probe method with an MCP-T370 (Nittoseiko Analytech Co., Ltd., Kyoto, Japan).

## 3. Results and Discussion

The evolution of 1# anode during the electrochemical (EC) process is presented in Figure 1a. At the beginning, at 10 min, black substances started to peel off from the open end of the anode and gathered on the upper layer of the electrolyte. The first segment gradually expanded and became a pocket-like shape due to the detachment of black substances. With time passing, the color of the electrolyte became darker, indicating that the quantity of the stripped substances increased significantly. At about 15 min, the first segment of tape automatically fell, and the electrolyte continued darkening, but details coul not be well observed (as seen in the photo taken at 90 min). At 130 min, the process was terminated, and substantial black products were collected in the beaker. As shown in Figure 1b, 1# anode finally turned into five parts: one was connected to the end and the other four fell into the electrolyte. The electric current–time curve (I-t, Figure 1c) reveals the EC process under the constant voltage of 10 V. The magnitude of the current reflects the intensity of electrochemical reactions between the electrolyte and graphite foil. The whole electrochemical process can be divided into five sections according the I-t curve. Every section shows a similar change trend, namely a rapid increase to a maximum peak followed by a slow decay. This current variation is caused by the change in the tape-wrapped anode during the electrochemical treatment. In the initial period of every section, gas expanded the graphite anode and increased the exposed area to electrolyte, which led to the rapid growth of current. Afterwards, the current decreased with the gradual depletion of the anode. The fifth section was terminated by a continuous current decay to about 0.1 A. The wrapped tape in this section could not detach, which restricted the electrochemical reactions to a low level due to the slow electrolyte transfer. It should be noted that the current was not interrupted during the whole process, which indicates the tape-wrapping electrochemical exfoliation was continuous, though the anode fell into five sections. The stripped products in the electrolyte were collected after washing, ultrasonication and centrifugation to prepare water dispersion (2 g/L). Further, graphene powder (0.583 g) and slurry (~20 g/L) were obtained after lyophilization and dispersing. Based on the easy-to assemble dispersion, a free-standing and flexible film was fabricated directly by vacuum filtration (Figure 1d).

### 3.1. Characterization of w-Gr

**Figure 2:**
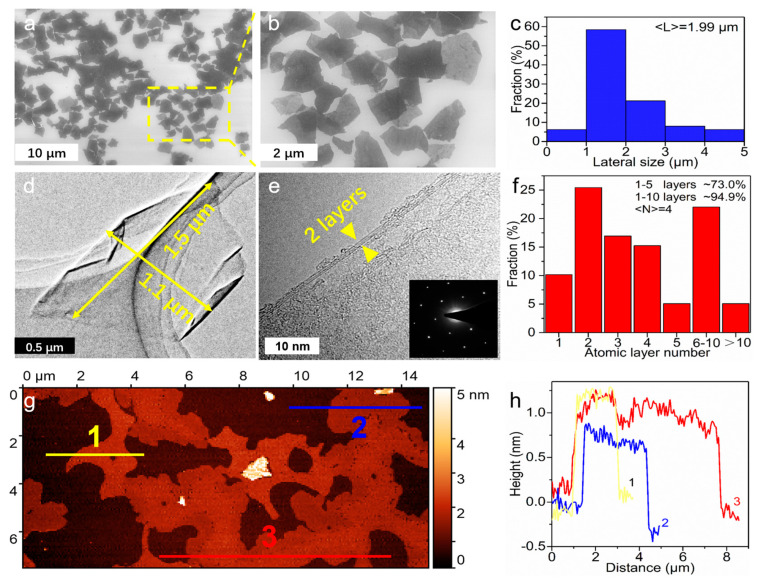
Morphology characterization of w-Gr. (**a**) Typical SEM image. (**b**) SEM image in the yellow square in (**a**). (**c**) Statistical distribution of lateral sizes from SEM image. (**d**) Typical TEM image of w-Gr. (**e**) HR-TEM image of w-Gr, inset shows the SAED patterns of w-Gr. (**f**) Statistical distribution of atomic layer numbers from TEM images. (**g**) Typical AFM image and (**h**) height profiles of w-Gr.

Scanning electron microscopy (SEM), transmission electron microscopy (TEM) and atomic force microscopy (AFM) [25] were used to characterize the morphology, thickness and size of the as-prepared product. Figure 2a presents the typical SEM image of the obtained sheets using 1# anode, and a high-magnification SEM image (Figure 2b) clearly shows these sheets are irregular in shape. The size distribution results obtained by SEM statistics data (Figure 2c) indicate that the average size of the sheets was 1.99 μm. Further, SEM-EDX analysis shows the element composition of w-Gr powder. The EDX spectra (Figure S4) confirm the existence of C and O, and the at.% of O accounts for 14.64% (Table S1). It is worth noting that no other elements were detected in the sample, which demonstrates that there were no impurities in the w-Gr products. Consistent with the SEM results, the TEM image in Figure 2d shows a typical graphene sheet with the size of 1.1 × 1.5 μm^2^. An HR-TEM image (Figure 2e) determines the bilayer feature of this sheet by clearly presenting the lattice stripe. The sixfold symmetric diffraction spots evidenced by selected area electron diffraction (SAED) patterns indicate that the as-prepared sheets possessed an *sp*^2^ crystal structure (Figure 2e and Figure S5). HR-TEM images with higher magnification (Figure S5a,b, scale bar: 5 nm) further evidence the crystal structure of w-Gr. An IFFT image (Figure S5c) presents single hexagonal carbon atom rings of the graphene structure, and an FFT image (Figure S5d) shows two sets of hexagonal spots, indicating two stacked crystal graphene sheets (about several atomic layers for each sheet) with different orientations [26,27]. The above TEM results suggest that the EC process well retained the high crystallinity of graphene. Further statistic data based on HR-TEM (Figure 2f and Figure S6) demonstrates that nanosheets with 1–5 atomic layers in products accounted for 73.0% and those with 1–10 layers accounted for 94.9%. The atomic layer number was averaged to be 4. An AFM image (Figure 2g) shows the as-prepared sheets with a thickness of about 1 nm (Figure 2h), indicating the presence of a single atomic layer [28].

**Figure 3:**
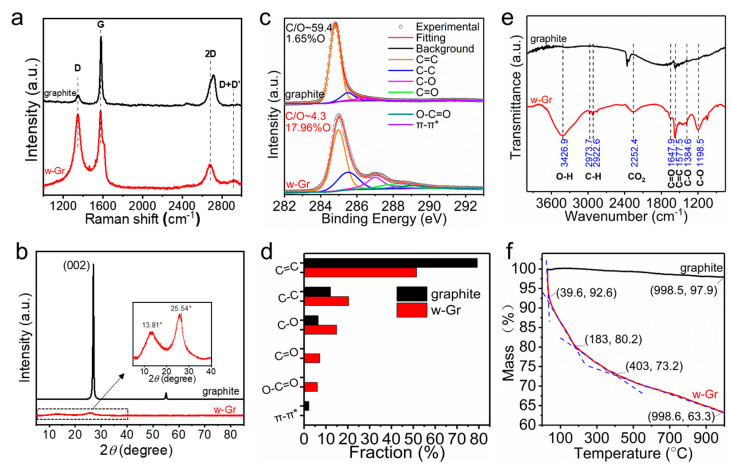
Structural characterization of w-Gr. (**a**) Raman spectra. (**b**) XRD spectra. (**c**) C1s XPS spectra. (**d**) Relative content of functional groups. (**e**) FT-IR spectra. (**f**) TGA profile.

Raman, X-ray diffraction (XRD), X-ray photoelectron spectroscopy (XPS), Fourier transform infrared spectrometry (FT-IR) and thermogravimetric analysis (TGA) were employed to analyze the change in microstructure and the chemical composition. Raman spectroscopy provides a great deal of valuable information in characterizing carbon materials, especially with respect to the carbon skeleton structure [29] and phonon vibrational modes inside and outside the surface [28]. The G peak (~1580 cm^−1^) reflected the Raman activity of high-frequency E_2g_ phonons triggered by the *sp*^2^ carbon network. The D peak (~1350 cm^−1^) included more complex information, such as disordered carbon, lattice defects and a particle size effect [30]. Figure 3a shows the distinct D (1346 cm^−1^), G (1575 cm^−1^) and 2D (2681 cm^−1^) peaks of the product. The ratio of peak intensity of D to G peaks (*I*_D_/*I*_G_) can be used to quantitatively evaluate the degree of defects and crystallinity [31]. The D peak intensity of w-Gr was enhanced compared to the precursor, and the *I*_D_/*I*_G_ increased from 0.12 to 0.96 as a result of the introduction of oxygen-containing functional groups. The lateral crystalline domain size (*L*a) [32] was calculated by Raman spectroscopy through a general formula involving the laser line wavelengths λ and *I*_D_/*I*_G_ (Figure S7). The decrease in *L*a reflects that the domain boundaries of sheets increased after EC treatment, which was contributed by the formation of new chemical bonds. Raman spectra provided direct evidence for the changes in graphite structure during the EC process. Moreover, the XRD spectra of raw graphite and w-Gr powder are shown in Figure 3b, and the interlayer space of (002) reflection (d_002_) was calculated using Bragg’s equation (n λ = 2d sin *θ*, where n = 1, λ of 0.1541 nm is the wavelength of Cu Kα1). Compared with graphite, w-Gr sheets showed a weak and wide peak centered at 25.54°, corresponding to a d_002_ of 0.3486 nm. The FWHM of this peak increased to 4.95°, which suggested a significant reduction in the sheet stacking along the c-axis during the electrochemical exfoliation, and the presence of w-Gr sheets with thickness more than one layer. The introduction of oxygen-containing functional groups may play an important role in increasing the interlayer space (from 0.3303 nm to 0.3486 nm). In addition, a peak was located at 2*θ* of 13.81°, which is ascribed to the diffraction peak of stacked w-Gr sheets. XRD results confirmed that the obtained products contained w-Gr sheets with different thicknesses. The chemical composition of the w-Gr was further identified by an XPS survey [33] (Figure S8). A narrow scan of C1s spectra (Figure 3c) shows a specific distribution of oxygen-containing groups for w-Gr. Five carbon peaks are attributed to the C=C peak (284.98 eV), the C-C peak (285.5 eV), the hydroxyl peak (C-OH, 287.0 eV), the carbonyl peak (C=O, 287.8 eV) and the carboxylic peak (O-C=O, 290.0 eV), respectively. The oxygen content of the as-prepared w-Gr increased from 1.65% to 17.96% compared with raw graphite. It is conducive to protecting the lattice structure of graphene by controlling the oxygen content below 20%. The relative contents of these bonds were calculated by deconvolution analysis of XPS C1s spectra (Figure 3d). The *sp*^2^ carbon (C=C) fraction is one of the important parameters to characterize the oxidation degree of graphene [34]. The C=C fraction of the product is 50%, which decreased by less than 30% compared with the precursor. This demonstrates relatively mild damage to the structure of graphene sheets during the EC process. The content of hydroxyl groups increased to 15%, and advanced carboxyl groups accounted for 6%. In addition to XPS, oxygen-containing functional groups could be detected using FT-IR. The FT-IR transmittance spectra (Figure 3e) show O-H stretching vibrations (3426.9 cm^−1^), a C=O stretching vibration (1647.9 cm^−1^), a C-O stretching vibration (1198.5 cm^−1^ and 1384.6 cm^−1^), as well as C-H (2922.6 cm^−1^ and 2973.7 cm^−1^) and C=C (1577.5 cm^−1^) from *sp*^2^ hybridization. These FT-IR features are consistent with XPS results, suggesting that the introduction of these polar groups affords graphene good dispersibility in water. To sum up, the slight disruption of the C=C conjugated structure and the temperate increase of the oxygen content demonstrate the advantages of the tape-wrapping strategy, that is, the introduction of proper hydrophilic functional groups on the graphene sheets to achieve good water dispersibility without overly destroying the crystalline structure. 

A certain proportion of carbon–oxygen bonds not only changes the structure of graphene, but also has an effect on its thermal stability [35]. Thermogravimetry analysis (TGA) curves are shown in Figure 3f. The total mass loss of w-Gr was 36.7%, which is higher than that of raw graphite (2.1%). The process of mass loss with the increase in temperature can be divided into four stages: First, the evaporation of interlayer adsorbed water at low temperatures (30–40 °C) brings about a 7.4% mass loss. When constantly increasing temperature to 183 °C, the decomposition of labile oxygen-containing functional groups (hydroxy group) is the main mass loss [36]. From 200 to 400 °C, the relatively slow mass loss of 7% can be ascribed to the decomposition process of the more stable oxygen-containing functional groups (epoxy groups and carbonyls) [37]. Finally, the unstable carbon in the carbon skeleton is thermally decomposed into CO and CO_2_ in the high temperature range of 400–1000 °C [38], which contributes a mass loss of 9.9%.

### 3.2. Assembly of w-Gr

**Figure 4:**
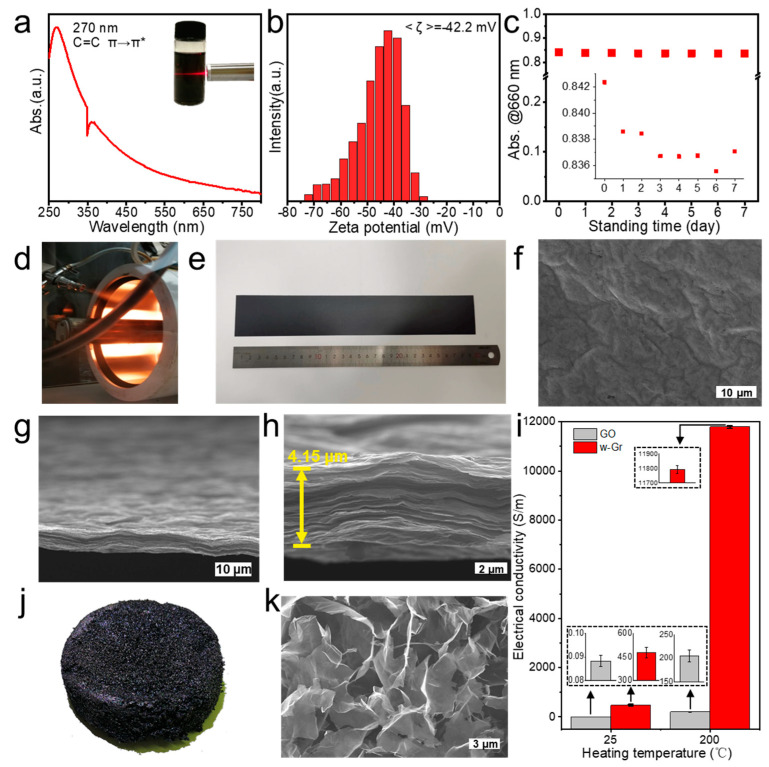
Assembly of w-Gr. (**a**) UV-vis spectrum of w-Gr aqueous dispersion with 4.5 mg/mL, inset shows the Tyndall effect. (**b**) Zeta potential of w-Gr aqueous dispersion (4.5 mg/mL). (**c**) Sedimentation behavior of w-Gr dispersion with 4.5 mg/mL. (**d**) Photograph of CCC process for preparing w-Gr films. (**e**) The w-Gr film with the size of 50 × 300 mm^2^. (**f**) Surface SEM image of the film. (**g**,**h**) Cross-section SEM images of the film. (**i**) Comparison of electrical conductivity for w-Gr film and GO film before and after thermal treatment (200 °C). (**j**) Photograph of w-Gr aerogels. (**k**) Typical SEM image of w-Gr aerogels.

Moreover, the dispersion of the as-prepared w-Gr was characterized to assess its water dispersibility. The inset in Figure 4a shows the Tyndall effect of the aqueous dispersion, indicating that the w-Gr dispersion resembles the dispersion state of colloids. However, the UV-vis spectrum (Figure 4a) of the 4.5 mg/mL dispersion shows that w-Gr exhibits a strong absorption peak at 270 nm, which is the π-π* transition of C=C bonds and indicates a well-retained conjugated structure of the dispersing sheets. Zeta potential is an important indicator to predict the dispersing stability of the colloid and explain the origin of stable dispersing. A high zeta potential (−42.2 mV, Figure 4b) was detected in an aqueous w-Gr dispersion of 4.5 mg/mL, which is more negative than −30 mV [39]. This large zeta potential verifies that sufficient electrostatic repulsion should be responsible for the excellent stability in water of the product [40]. To further analyze the sedimentation behavior of the dispersion, we recorded the intensity of the UV-vis absorption peak at 660 nm of the aqueous dispersion (Figure 4c). The absorbance of the w-Gr aqueous dispersion was 0.842 in the very beginning, and decreased to 0.837 after 7 days of sedimentation. The 0.6% decrease can be ascribed to the flocculation and sedimentation of the sheets. The relatively constant value of absorbance over a week suggests a high stability of the w-Gr dispersion with a high concentration (~4.5 mg/mL). The w-Gr powder freeze-dried over a month ago was redispersed into water to prepare for dispersion with a concentration of 4.5 mg/mL. It can be easily redispersed in water with the aid of mild sonication (30 min). The zeta potential was measured to estimate the stability of the nanosheets. The result shows an average zeta potential of −42.3 mV (Figure S9), which is consistent with that of dispersed freshly freeze-dried w-Gr (−42.2 mV). Therefore, the w-Gr nanosheets exposed to air for more than 4 weeks still possessed good water dispersity. We continued to let them stand for half a month to evaluate their long-term stability, and measured the zeta potentials every five days. No apparent flocculation was observed but a grey layer appeared on the surface (Figure S9). The zeta potentials presented a decrease trend to −36.7 mV in half a month, indicating the decayed stability. This can be ascribed to the solvent evaporation, as revealed by the gradually declining liquid level (Figure S9) due to direct exposure to air, which led to increased concentration and decreased stability [41]. The as-prepared w-Gr, as one of the accessible precursors, addresses the difficulty of direct assembly of graphene due to its hydrophobicity. It can be easily and stably dispersed in water rather than organic solvents without the assistance of extra surfactant. On the other hand, unlike GO [42,43,44,45], w-Gr has a lower degree of oxidation and a proper proportion of oxygen groups, and retains most of the *sp*^2^ domains and expresses an advantage over the normal GO in terms of structure restoration. Moreover, the oxygen-containing functional groups in w-Gr can be removed under low thermal annealing (below 1000 °C). The high-temperature graphitization treatment is not required, which simplifies the preparation process and reduces the energy consumption of production [46]. We demonstrate the use of w-Gr as a precursor to prepare graphene films (Figure 4e) and porous aerogels (Figure 4j), which are typical macroscopic assemblies of GO [47,48]. The preparative process of w-Gr film by a CCC system is shown in Figure 4d, which will enable the scalable production of conductive films. SEM images (Figure 4f,g) reveal that the obtained w-Gr film (Figure 4e) was constructed by numerous stacked sheets with ripples. The thickness of the film is about 4.15 μm (Figure 4h). After thermal annealing at 200 °C for 1 h, the electrical conductivities of w-Gr film and GO film increased by 25 and 2323 times, respectively (Figure 4i), which suggests that oxygen-containing functional groups can be effectively removed through thermal annealing. However, w-Gr film exhibited an average electrical conductivity of 11,793 S/m, which is 58 times more than GO film (205 S/m). This demonstrates that w-Gr well preserves the lattice structure of graphene. A typical SEM image of the w-Gr aerogel (Figure 4k) shows a porous network constructed by interconnected rippled sheets. The density was measured to be 9.8 mg/cm^3^. 

### 3.3. Mechanism Analysis

**Figure 5:**
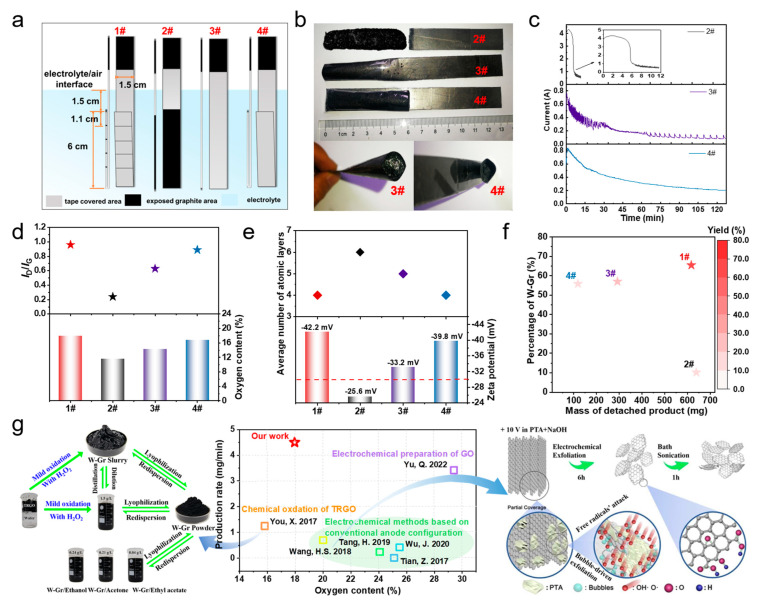
Comparison of 1–4# anodes. (**a**) Illustration of 4 types of anodes for tape-wrapping approach. (**b**) Photographs of 2–4# anodes after the termination of electrochemical reaction and open end of 3# and 4# anodes. (**c**) I-t curves from 0 to 120 min. (**d**) Oxygen content and *I*_D_/*I*_G_ of w-Gr. (**e**) Zeta potentials of w-Gr dispersion and average number of atomic layers of w-Gr. (**f**) Yield of w-Gr. (**g**) Advantages of tape-wrapping strategy [20,22,23,39,49,50].

It should be pointed out that the key to successful preparation of w-Gr lies in the design of anode configuration. The proposed tape-wrapping strategy makes possible the mild yet sufficient oxidation of graphite during exfoliation. On this basis, we make efforts to optimize the wrapping configuration to maximize yield and efficiency. In fact, 1# anode is the result of optimization. Here, we compare it with 2–4# anodes to explore the possible mechanism underlying the efficient preparation of w-Gr. The exfoliation area of 2# anode is not wrapped by tape, which is the same as conventional electrochemical methods, whereas 3# and 4# anodes are wrapped by a whole tape without segmentation. The open end of 3# anode is downward while that of 4# anode is upward (Figure 5a). 

Firstly, the electrochemical processes of different anodes were recorded (Figure S10), and apparently different phenomena were found. At the initial 10 min, a large amount of gas was produced on the surface of 2# anode by the electrolysis of water, and a mass of black substances peeled off from the anode rapidly. The electrolyte soon darkened; it was hard to see straight-on the change in the anode. The final as-obtained products consist of black powders and a whole detached electrode (Figure 5b). This suggests that the physical destruction of the anode by the bubbles in the absence of tape wrapping results in the inability of the EC process to proceed uniformly. The EC process of 3# and 4# anodes seemed to be slowed down by tape wrapping because the detachment of products proceeded in a milder way. For 3# anode, the eventual formation and release of a big bubble at the downwards end dominated the last term of the reaction progress. At the end of the EC process, 3# and 4# anodes turned out to be pocket-filled with black slurry (Figure 5b), and the colors of the two electrolytes were apparently lighter than that of 2#. I-t curves (Figure 5c) give more details about the EC process. The I-t curve indicates that the EC reaction on 2# anode almost ended within 8 min, which was the fastest one among the four cases, and was consistent with the rapid depletion of the anode. The current of 3# anode was 0.8 A at the beginning of the reaction because only the bottom was exposed to the electrolyte. In the first 30 min, the current decreased to about 0.2 A as the sheets gradually detached from the bottom end. In 30 to 70 min, the release of bubbles was delayed, which slowed the decrease in current. After 70 min, the oscillation of the current synchronized with the accumulation and burst of big bubbles. This may be ascribed to the impeded mass transfer and electrochemical processes by the formation of gas inside the tape. The current of 4# anode reduced from the initial 0.8 A to 0.5 A within only 20 min. This trend is consistent with that of 1# anode, where it was in the range of 0–15 min. However, the current changed slowly thereafter and remained at around 0.2 A in the end. The accumulation of products in the tape cavity was considered to be the main reason for the unsustainability of the EC process. It is evident that none of the above three types of anode configuration can achieve a sufficient and continuous EC process.

Further, Raman, XPS and AFM were also carried out to analyze the structure, chemical composition and sheet thicknesses of the water-dispersible products (w-product). The oxygen content and the ratio of peak intensity of D to G peaks (*I*_D_/*I*_G_) reflect the degree of oxidation for different w-products. Zeta potentials are an indicator for the stability of aqueous dispersions, and a value more negative than −30 mV is considered necessary for stable dispersing [39]. These parameters for different samples are presented in Figures S11–S13 and compared in Figure 5d,e. Compared with conventional anode configuration (2#), tape wrapping (1, 3, 4#) was proven effective in increasing the water dispersibility by ensuring adequate oxidation. The zeta potential (|ζ|) was proportional to the oxygen content of the w-Gr sheets (Figure 5d,e), which indicated that w-Gr dispersion is more stable due to the deprotonation of the oxygen-containing functional groups contributing to the greater charge of the w-Gr sheets [51]. The conductivity of the samples in DI water was simultaneously evaluated during the zeta potential measurement. As shown in Table S3, it was presented that the conductivity of the 1# w-Gr dispersion reduced to 0.00812 mS/cm compared to that of 2#, which may be ascribed to the effect of surface charge by the increase in oxygen functional groups. Also, a lower average number of atomic layers (Figure 5e) was found in w-products of the three tape-wrapped anodes, and the optimized wrapping configuration (1#) produced the thinnest sheets, which suggests the importance of tape wrapping in enhancing electrochemical exfoliation. It should be noted that the effect of tape wrapping configuration on water dispersibility was similar to that of the sheet thickness. This finding highlights the importance of tape wrapping and specific wrapping configuration in enhancing both electrochemical oxidation and exfoliation, which are equally critical for the fabrication of w-Gr. Therefore, the differences in the products of 1#, 3# and 4# anodes indicate that the wrapping configuration should be given priority and optimized. Moreover, we made more analyses to calculate the yield and production rate of w-Gr (Table S3). Sheets with atomic layers less than or equal to 5 in water-dispersible products are defined as w-Gr. Figure 5f shows the yields of w-Gr obtained by multiplying the mass of the detached product with the percentage of w-Gr. Although the mass of the detached product for 2# anode was the largest (0.639 g), w-Gr accounted for only 10.16% of the product. 1# anode with segmental wrapping had the highest yield (65.44%) of w-Gr. As for the production rate of w-Gr, 1# anode was surpassed by 2# anode because of the much faster EC process. However, considering the much lower mass of w-Gr in every batch and the instability of the w-Gr (ζ = −25.6 mV), 1# anode is the most suitable wrapping configuration for large-scale preparation. The above product characterization results and mechanism analysis clearly demonstrate the features of the tape-wrapping strategy. In order to further illustrate the advantages of this method, it is necessary to compare it with previous methods (Figure 5g). The tape-wrapping strategy proposed in this work belongs to the optimization of anode configuration in the electrochemical methods rather than to molecule anode coverage (Figure 5g). Compared with the chemical oxidation of thermally reduced graphene oxide (TRGO) [39] and existing electrochemical methods based on conventional anode configuration [22,23,49,50], the tape wrapping strategy of fabricating w-Gr focused on the balance of proper oxidation and deep exfoliation during the continuously electrochemical process, and controlled the relatively low oxygen content (17.96%) while enabling the good water dispersibility of graphene, which was quite different from that of the GO [20]. Meanwhile, the tape-wrapping strategy significantly improved the production rate to 4.5 mg/min, which is six times faster than the maximum of previous electrochemical methods of w-Gr. Therefore, this green electrochemical method represents an important breakthrough in terms of the preparation efficiency of w-Gr, which is desirable for massive preparation. 

In the conventional EC process, the process of intercalation, oxidation and exfoliation generally overlaps rather than proceeds in a sequence [52], and even the exfoliation by gas bubbles reacts priorly to the oxidation, leading to the detachment of the anode in advance [41]. Actually, this problem exists widely in the currently mature methods of electrochemical exfoliation for the preparation of graphene. In this work, tape wrapping provides a strategy featuring the physical limitation of the damage of anodes by bubbles. From the above analyses of the phenomena and I-t curves of the EC processes as well as the corresponding products, we can roughly get the mechanism of our optimized tape-wrapping strategy: physical confinement by tape wrapping prolongs the time that electrochemically generated oxidative substances take to act on graphene to afford oxygen groups and water dispersibility. More importantly, wrapping configuration optimization by segmentation allows the automatic detachment of each section and avoids suppressed mass transfer because of the physical confinement, and, thus, ensures the sustainability of the slowed EC process.

## 4. Conclusions

In summary, we proposed a tape-wrapping strategy to electrochemically prepare few-layered water-dispersible graphene. It was demonstrated that the optimized wrapping configuration realizes w-Gr fabrication with a yield of 65.44% and a production rate of up to 4.5 mg/min. Further mechanism analysis suggests that tape wrapping ensures the proper oxidation and deep exfoliation of graphite while segmentation balances both the quality and efficiency of products. The rational design of anode configuration plays a crucial role in ensuring a uniform and continuous electrochemical process. This method is carried out in a green system with high efficiency, which can provide new methodological guidance for the industrial preparation of graphene.

## Data Availability

Data are contained within the article.

## Supplementary Materials

The following supporting information can be downloaded at: https://www.mdpi.com/article/10.3390/nano14090805/s1. Figure S1: Photographs of electrochemical DC power source and reaction equipment (**a**), and the four anode configurations (**b**,**c**). Figure S2: Three-dimensional model of the four anode configurations. Figure S3: Photograph of vacuum filtration setup for preparing w-Gr films. Figure S4: (**a**) EDX analysis of w-Gr. (**b**) SEM image, EDX mapping showing the C (**c**) and O (**d**) distribution on w-Gr surface. Figure S5: (**a**) Typical HR-TEM image of 1# w-Gr sheet (Scale bar: 5 nm). (**b**) HR-TEM image in the yellow rectangle (Scale bar: 1 nm). (**c**) Corresponding IFFT image and (**d**) FFT image in the yellow rectangle. Figure S6: Typical HRTEM patterns of 1# w-Gr at folded edges with different atomic layer numbers (Scale bars in **a**–**f**: 10 nm). Figure S7: The comparison of structure parameters of graphite and w-Gr determined by Raman. Figure S8: The XPS survey spectra of graphite and w-Gr. Figure S9: Photographs and zeta potentials of w-Gr dispersion taken every five days. Figure S10: EC process of 2-4# anodes in 1 M (NH_4_)_2_SO_4_ (from top to the bottom). 0 minute (**a**,**e**,**i**), 10 minutes (**b**,**f**,**j**), 15 minutes (**c**,**g**,**k**), and 90 minutes (**d**,**h**,**l**). Figure S11: Raman spectra of w-products for 2-4# anodes. Figure S12: The thickness distribution (**a**,**d**,**g**), C1s XPS spectra (**b**,**e**,**h**), and zeta potential (**c**,**f**,**i**) of w-products for 2-4# anodes. Figure S13: The typical AFM images of w-Gr prepared from 2-4# anodes. Table S1: Elemental analysis of w-Gr based on the EDX results. Table S2: The comparison of structure parameters of graphite and w-Gr determined by XRD. Table S3: Calculation of the yield and production rate of w-Gr for 1-4# anodes. Table S4: Calculation of the yield and production rate of w-Gr for 1-4# anodes. Video S1: Dispersibility of w-Gr.
