# Supplementary material for: A Tape-Wrapping Strategy towards Electrochemical Fabrication of Water-Dispersible Graphene
Source: Nanomaterials (Basel). 2024 May 6;14(9):805. doi: 10.3390/nano14090805 (PMC11085361; doi:10.3390/nano14090805)
Supplement: Supplementary file 1 [file nanomaterials-14-00805-s001.zip › Supporting Information-nanomaterials-2986798.pdf]

A Tape-Wrapping Strategy towards Electrochemical Fabrication of Water-Dispersible Graphene

Deyue Xiao [1,2], Peng He [1,2,*], Haolong Zheng [1,2], Shujing Yang [1,2], Siwei Yang [1,2] and Guqiao Ding [1,2,*]

1 National Key Laboratory of Materials for Integrated Circuits, Shanghai Institute of Microsystem and Information Technology, Chinese Academy of Sciences, 865 Changning Road, Shanghai 200050, China
2 College of Materials Science and Opto-Electronic Technology, University of Chinese Academy of Sciences, Beijing 100049, China
* Correspondence: hepeng@mail.sim.ac.cn (P.H.); gqding@mail.sim.ac.cn (G.D.)

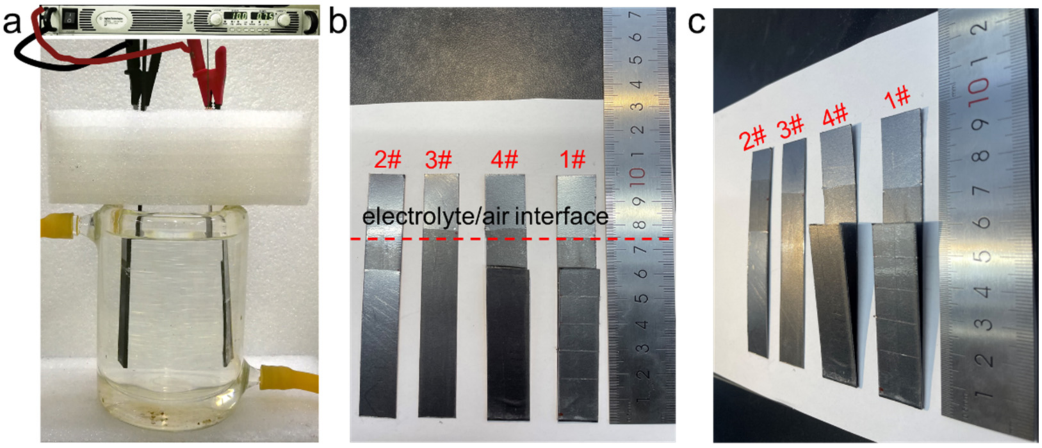

Figure S1. Photographs of electrochemical DC power source and reaction equipment (a), and the four anode configurations (b-c).

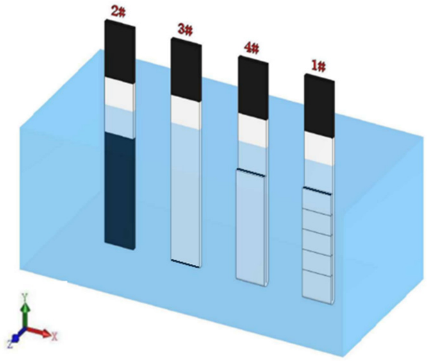

Figure S2. Three-dimensional model of the four anode configurations.

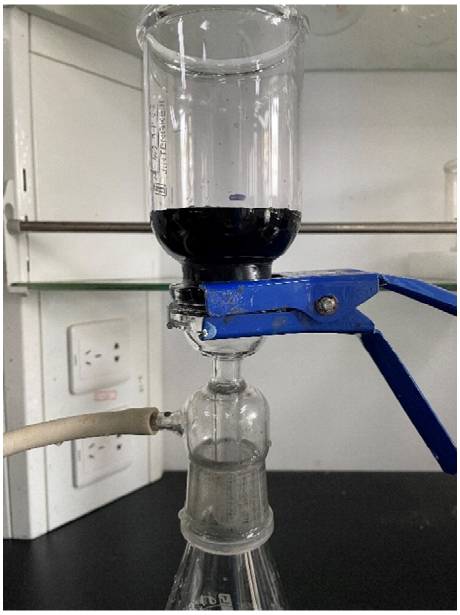

Figure S3. Photograph of vacuum filtration setup for preparing w-Gr films.

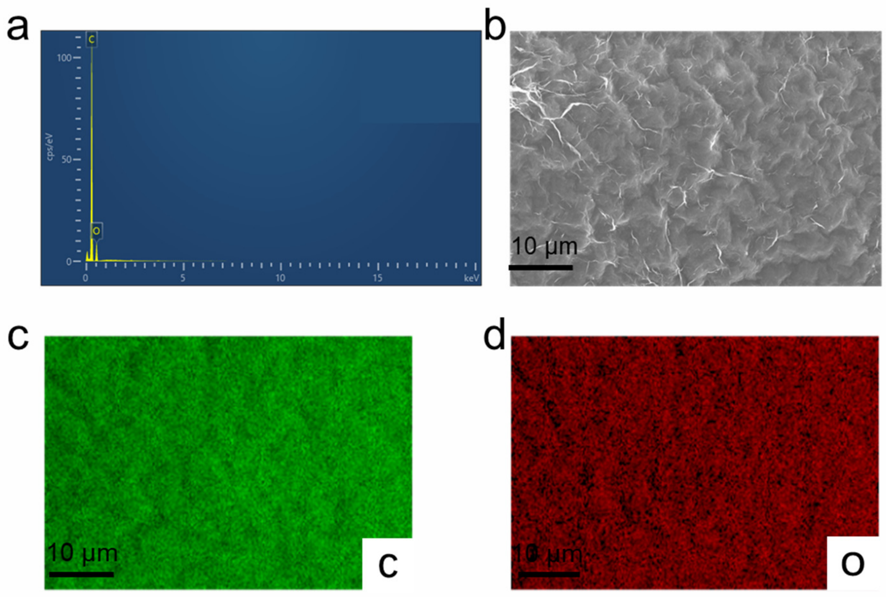

Figure S4. (a) EDX analysis of w-Gr. (b) SEM image, EDX mapping showing the C (c) and O (d) distribution on w-Gr surface.

Table S1. Elemental analysis of w-Gr based on the EDX results.

Element	Content (wt.%)	Content (at.%)
C	81.40	85.36
O	18.60	14.64

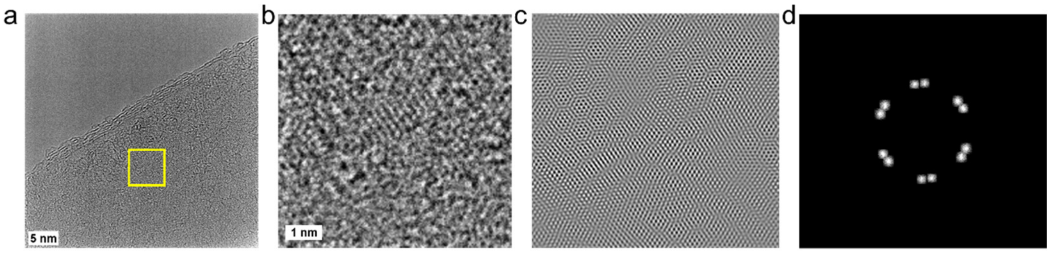

Figure S5. (a) Typical HR-TEM image of 1# w-Gr sheet (Scale bar: 5 nm). (b) HR-TEM image in the yellow rectangle (Scale bar: 1 nm). (c) Corresponding IFFT image and (d) FFT image in the yellow rectangle.

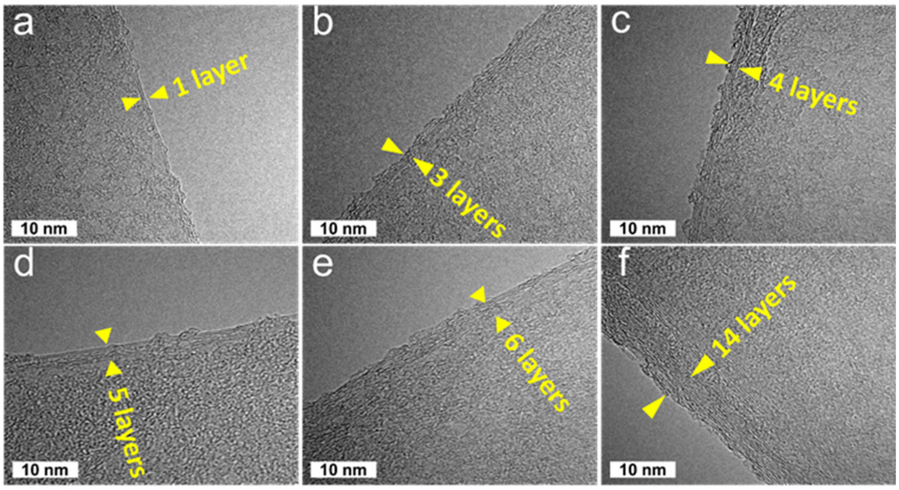

Figure S6. Typical HRTEM patterns of 1# w-Gr at folded edges with different atomic layer numbers. (Scale bars in a–f: 10 nm)

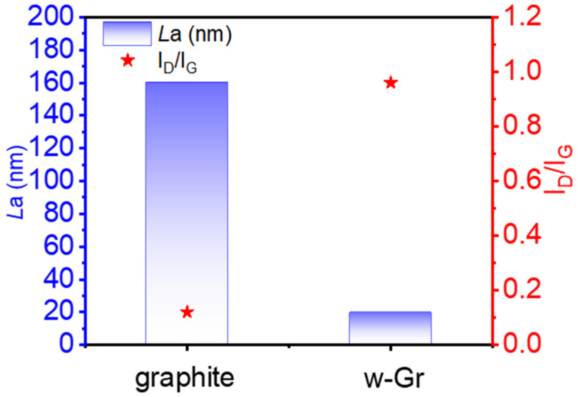

Figure S7. The comparison of structure parameters of graphite and w-Gr determined by Raman.

Table S2. The comparison of structure parameters of graphite and w-Gr determined by XRD.

(002)	Graphite	w-Gr
2θ (degree)	26.97	25.54
$d_{(002)}$ (nm)	0.3303	0.3486
FWHM (degree)	0.41	4.95

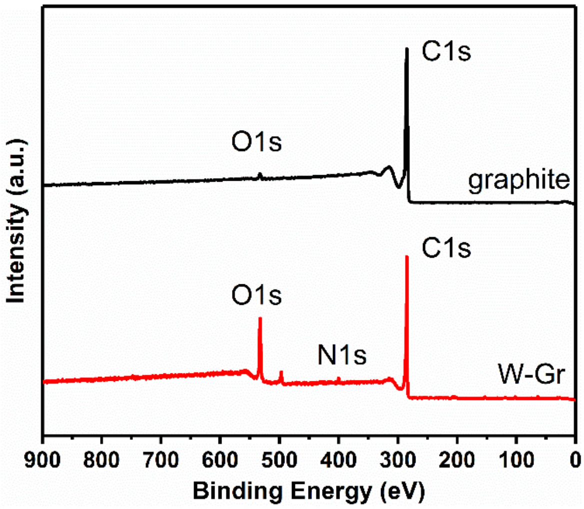

Figure S8. The XPS survey spectra of graphite and w-Gr.

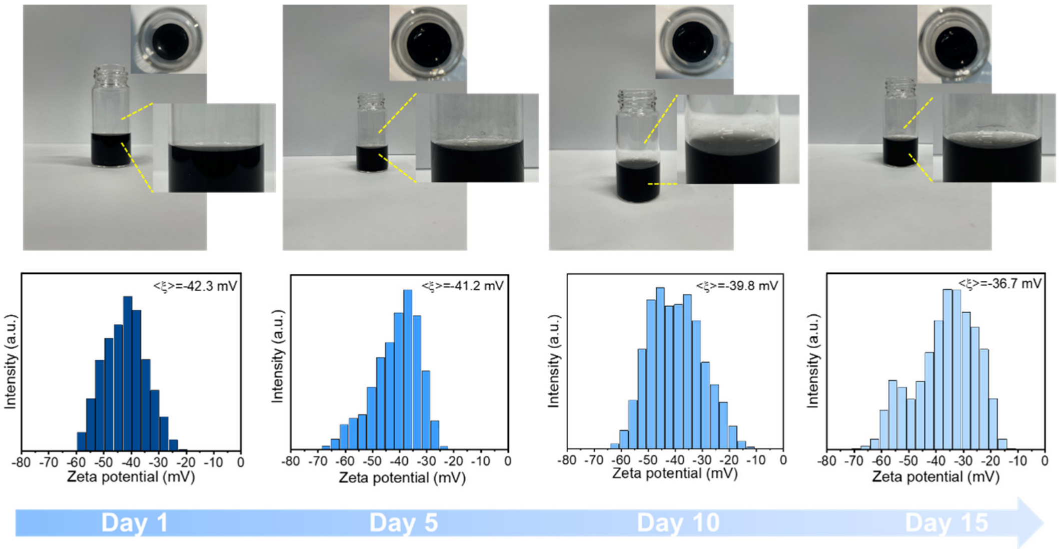

Figure S9. Photographs and zeta potentials of w-Gr dispersion taken every five days.

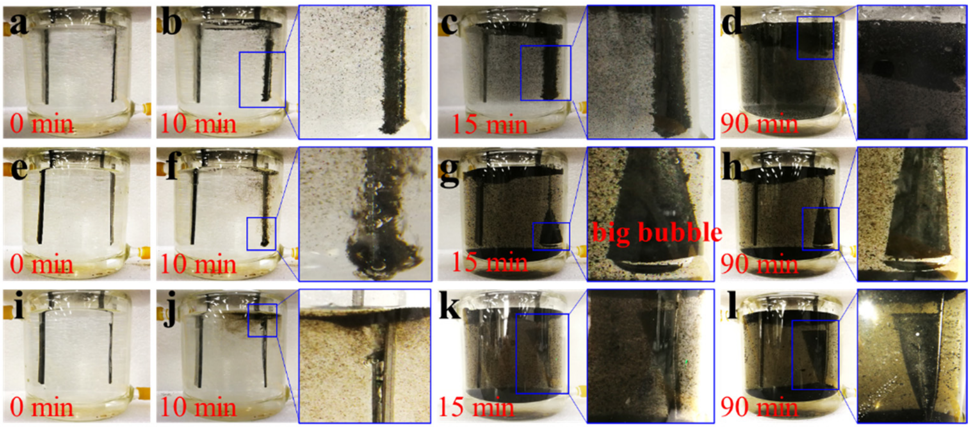

Figure S10. EC process of 2-4# anodes in 1 M $(NH_4)_2SO_4$ (from top to the bottom). 0 minute (a, e, i), 10 minutes (b, f, j), 15 minutes (c, g, k), and 90 minutes (d, h, l).

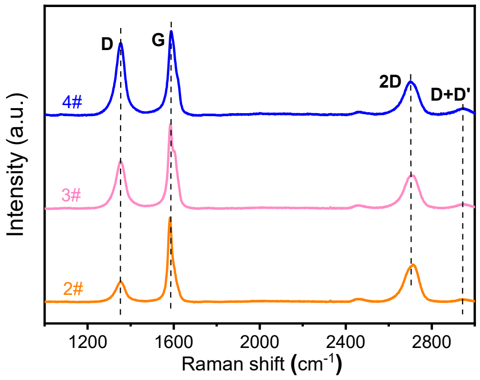

Figure S11. Raman spectra of w-products for 2-4# anodes.

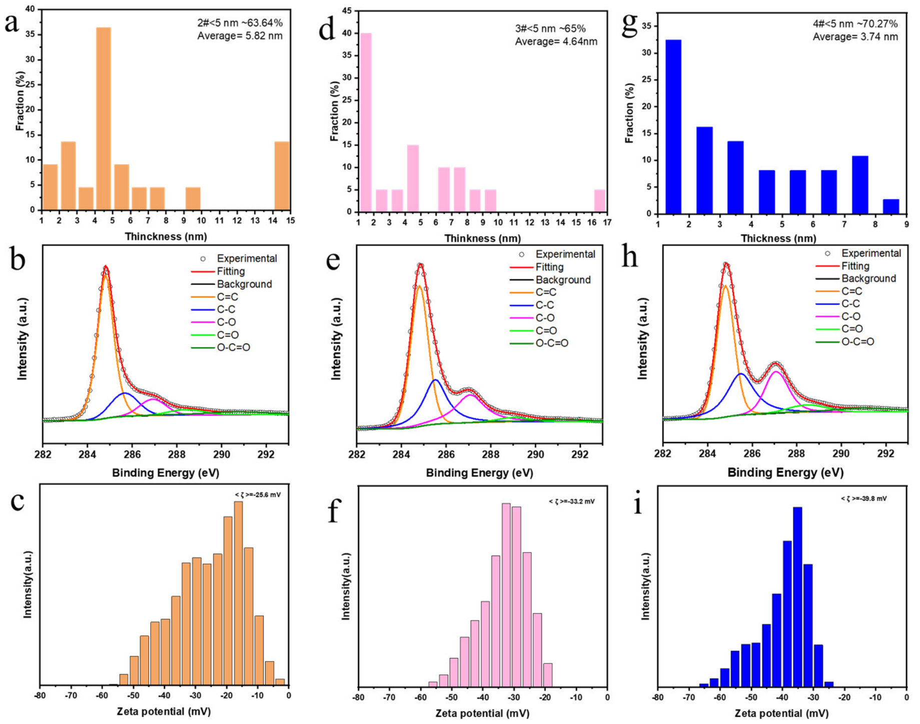

Figure S12. The thickness distribution (a, d, g), C1s XPS spectra (b, e, h), and zeta potential (c, f, i) of w-products for 2-4# anodes.

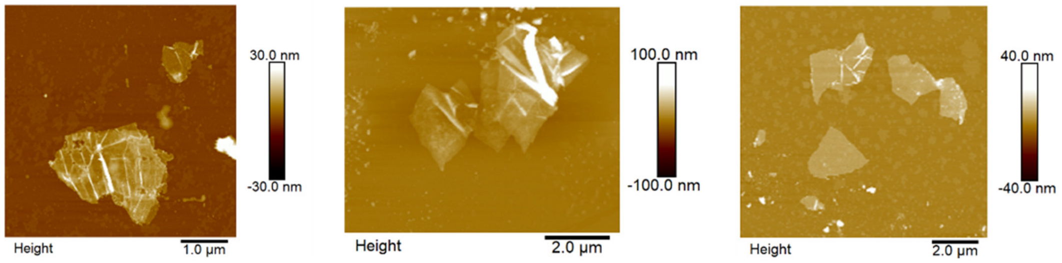

Figure S13. The typical AFM images of w-Gr prepared from 2-4# anodes.

Table S3 Calculation of the yield and production rate of w-Gr for 1-4# anodes.

Sample	Zeta potential (mV)	Conductivity (mS/cm)
1#	42.2	0.00812
2#	25.6	0.0103
3#	33.2	0.0125
4#	39.8	0.00738

Table S4. Calculation of the yield and production rate of w-Gr for 1-4# anodes.

**NO.**	**Configuration of anode**	**Electrochemical period (min)**	**Mass of product detached from the anode (g)**	**Mass of water-dispersible product (g)**	**Percentage of water-dispersible product (%)**	**Percentage of graphene in water-dispersible product (%)**	**Percentage of water-dispersible graphene in detached product (%)**	**Mass of water-dispersible graphene in one batch (mg)**	**Yield (%) =M(w-Gr)/M(raw graphite)**	**Everage time of anode exfoliation (min/g)**	**Production rate of water-dispersible graphene (mg/min)**
1#	side faces segregationally wrapped and folded with open end upwards	90	0.617	0.554	89.79	72.88	65.44	403.763	65.44	145.867	4.486
2#	side faces and end unwrapped	8	0.639	0.102	15.96	63.64	10.16	64.913	10.52	12.520	8.114
3#	side faces wrapped with open end downwards	70	0.292	0.256	87.67	65.00	56.99	166.400	26.97	239.726	2.377
4#	Folded with side faces wrapped and end open upwards	120	0.118	0.0937	79.41	70.27	55.80	65.843	10.67	1016.949	0.549
